# Autoimmune glomerulonephritis in a multiple sclerosis patient after cladribine treatment

**DOI:** 10.1177/13524585211022719

**Published:** 2021-06-24

**Authors:** Kristina Schönfelder, Helene Schuh, Frederick Pfister, Julia Krämer, Ute Eisenberger, Jelena Skuljec, Jana Hackert, Tobias Ruck, Steffen Pfeuffer, Michael Fleischer, Anja Gäckler, Tim Hagenacker, Andreas Kribben, Sven G Meuth, Christoph Kleinschnitz, Refik Pul

**Affiliations:** Department of Nephrology, University Medicine Essen, Essen, Germany Center for Translational Neuro- and Behavioral Sciences, University Hospital Essen, Essen, Germany; Department of Neurology, University Medicine Essen, Essen, Germany/Center for Translational Neuro- and Behavioral Sciences, University Hospital Essen, Essen, Germany; Department of Nephropathology, University Medicine Erlangen, Erlangen, Germany; Department of Neurology with Institute of Translational Neurology, University Hospital Münster, Münster, Germany; Department of Nephrology, University Medicine Essen, Essen, Germany Center for Translational Neuro- and Behavioral Sciences, University Hospital Essen, Essen, Germany; Department of Neurology, University Medicine Essen, Essen, Germany/Center for Translational Neuro- and Behavioral Sciences, University Hospital Essen, Essen, Germany; Department of Neurology, University Medicine Essen, Essen, Germany/Center for Translational Neuro- and Behavioral Sciences, University Hospital Essen, Essen, Germany; Department of Neurology with Institute of Translational Neurology, University Hospital Münster, Münster, Germany; Department of Neurology with Institute of Translational Neurology, University Hospital Münster, Münster, Germany; Department of Neurology, University Medicine Essen, Essen, Germany/Center for Translational Neuro- and Behavioral Sciences, University Hospital Essen, Essen, Germany; Department of Nephrology, University Medicine Essen, Essen, Germany Center for Translational Neuro- and Behavioral Sciences, University Hospital Essen, Essen, Germany; Department of Neurology, University Medicine Essen, Essen, Germany/Center for Translational Neuro- and Behavioral Sciences, University Hospital Essen, Essen, Germany; Department of Nephrology, University Medicine Essen, Essen, Germany Center for Translational Neuro- and Behavioral Sciences, University Hospital Essen, Essen, Germany; Department of Neurology with Institute of Translational Neurology, University Hospital Münster, Münster, Germany; Department of Neurology, University Medicine Essen, Essen, Germany/Center for Translational Neuro- and Behavioral Sciences, University Hospital Essen, Essen, Germany; Department of Neurology, University Medicine Essen, Essen, Germany/Center for Translational Neuro- and Behavioral Sciences, University Hospital Essen, Essen, Germany

**Keywords:** Relapsing multiple sclerosis, cladribine tablets, adverse event, secondary autoimmunity, anti-glomerular basement membrane antibodies, thrombocytopenia, autoimmune hepatitis

## Abstract

**Background:**

Oral cladribine is an approved disease-modifying drug for the treatment of relapsing multiple sclerosis. In controlled clinical trials as well as in post marketing safety assessments, autoimmune conditions have not yet been reported as a specific side effect of cladribine.

**Objective and Results:**

Here, we report a case of anti-glomerular basement membrane antibody-mediated glomerulonephritis that occurred shortly after the fourth cladribine treatment cycle.

**Conclusion:**

Neurologists should be attentive to the development of secondary autoimmunity in cladribine-treated patients.

## Introduction

In Germany, cladribine tablets (CT; Cladribine, Merck, Darmstadt, Germany; Mavenclad®) are approved for the treatment of relapsing multiple sclerosis (RMS) since 2017. As an adenosine deaminase–resistant purine nucleoside, it selectively depletes T and B cells since these cells exhibit a high intracellular ratio of deoxycytidine kinase to deoxynucleotidase.^1^ Following treatment, T and B cells gradually deplete, reaching a nadir at month 4 and 2, respectively.^2^ Although the exact mechanisms remain elusive, the subsequent repopulation that results in altered immune properties probably relates to the therapeutic efficacy of cladribine.^3^ An increase of naïve B cell and M2 macrophage with a reduction of memory B and T cell counts has been reported at 2 years after treatment initiation.^4^ Autoimmune conditions have been reported neither in the pivotal studies (CLARITY, CLARITY Extension, ONWARD, ORACLE) nor in the PREMIERE registry or in the periodic safety update reports.^5–9^ Here, we present the first case of secondary autoimmunity in a multiple sclerosis (MS) patient under treatment with CT.

## Case report

**Figure 1.**
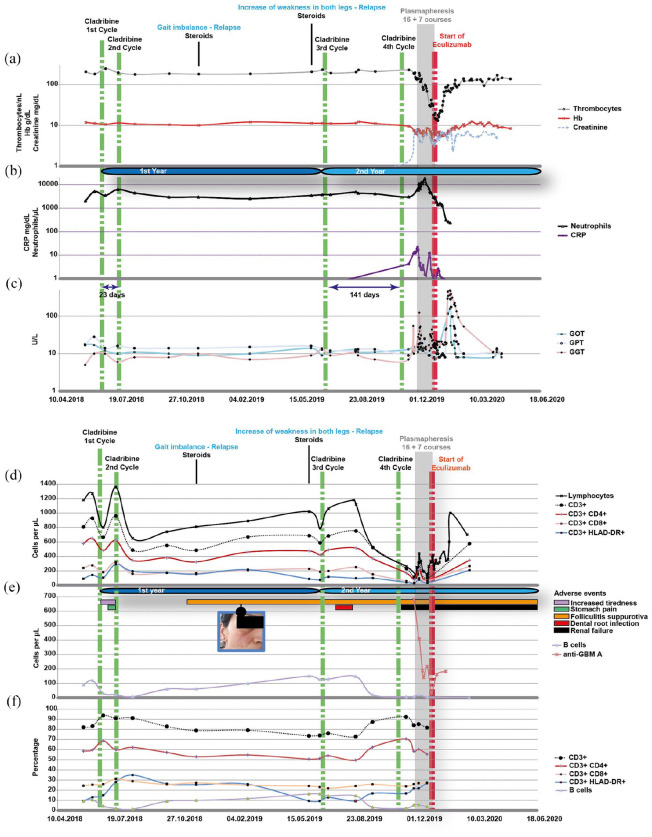
Overview about disease-related and immunological parameters upon cladribine treatment. (a) Decrease of hemoglobin (Hb; min. value 4.7 g/dL) and thrombocytes (min. value 13 platelets/nL) while serum creatinine values were increasing. Haptoglobin levels were constantly <0.01 g/L and increased in May 2020 to 0.35 g/L (data not shown). (b) Increase of C-reactive protein (CRP) was associated with hemolytic anemia, thrombocytopenia, and renal failure. Increase of leukocytes and neutrophils occurred after steroid administration followed by a decrease to 240 neutrophils per µL necessitating the application of granulocyte colony stimulating factor. (c) Increase of liver enzymes after the fourth cladribine course. GOT: aspartate aminotransferase; GPT: alanine aminotransferase; GGT: gamma-glutamyl transferase. (a)–(c) A base-10 log scale is used for the Y axis. (d) Lymphocyte and T cell subset (total CD3+ cells, CD3+ CD4+, CD3+ CD8+, and CD3+ HLADDR+ subsets) pharmacodynamics reveal a distinct decrease of lymphocytes following the third cladribine cycle. The slight drop and subsequent increase of lymphocyte count shortly after the first week of cladribine treatment in year 1 and 2 is associated with steroid treatments due to multiple sclerosis relapse. (e) Despite B cell counts close to zero, high levels of anti-glomerular basement membrane (GBM) antibodies were detected which decreased with plasma exchange therapy. (f) Proportional representation of immune cell subsets. Treatment courses, relapses, and treatment for anti-GBM glomerulonephritis are shown in (a) and (d). The chronology of adverse events is illustrated in (e).

**Figure 2.**
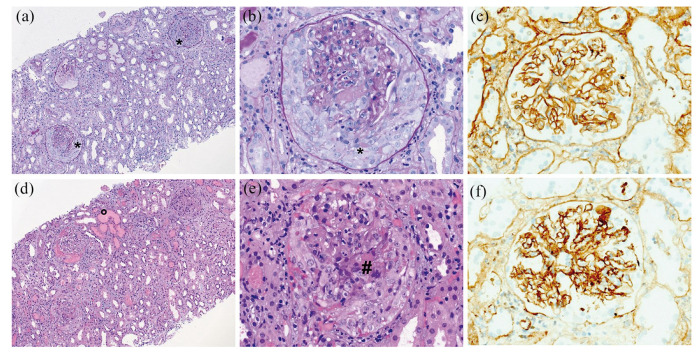
Light microscopy findings and immunohistochemical results of diffuse extracapillary proliferative and necrotizing anti-GBM-glomerulonephritis. (a) and (b), (d) and (e): Glomerulonephritis with diffuse cellular crescent formation (*), fibrinoid necrosis (#), accompanying acute tubular necrosis, erythrocyte casts (°), interstitial edema, and inflammation ((a) and (b): PAS-staining; (d) and (e): HE-staining). (c) and (f): Immunohistochemistry shows linear staining of the glomerular basement membrane with IgG (c) and C3c (f). Histological stainings for IgA, IgM, and C1q were negative (not shown). Magnification 100× in (a) and (d) and 400× in (b), (c), (e), and (f).

In a 44-year-old female patient with RMS, CT treatment course (year 1) was commenced in June 2018 (first cycle) after a spinal relapse (Figure 1(a)). Apart from migraine and arterial hypertension, the patient had no other comorbidities. As concomitant medication, she was taking fampridine 10 mg BID and amlodipine 5 mg QD. She was treated with interferon β-1a from June 2006 to March 2015 and with teriflunomide from March 2015 to June 2018. Prior to CT treatment, teriflunomide was washed out with colestyramine 3×8 g daily for 11 consecutive days. In year 2, the first CT cycle was started in June 2019 and the second one had been postponed because of a dental root infection, which was treated endodontically and with amoxicillin (Figure 1(e)). Two relapses occurred during year 1 requiring intravenous steroid administration (Figure 1(a)). Several adverse events such as increased tiredness and folliculitis occurred upon cladribine treatment (Figure 1(e)). In November 2019, the patient presented with fever and pollakisuria. Pyelonephritis associated with acute kidney injury (AKI) was suspected (Figure 1(a) and (b)). Despite ciprofloxacin treatment, the patient developed acute renal failure with oliguria, edema, and hypertension after 10 days, leading to hospitalization and eventually to hemodialysis. Kidney biopsy revealed a necrotizing glomerulonephritis with anti-glomerular basement membrane (GBM) antibodies (Figure 2). Anti-GBM antibodies were also detected in the serum with ⩾680 U/mL (Figure 1(e)). Steroid pulse therapy and plasmapheresis were initiated. Three weeks later, the patient developed bicytopenia with severe thrombocytopenia (18,000/µL) and a Coombs negative hemolytic anemia (Figure 1(a)). Bone marrow biopsy was without pathological findings. Laboratory examinations for Epstein–Barr virus, hantavirus, influenza (A and B), respiratory syncytial virus, cytomegalovirus, and HIV were negative. Because of distinct anemia, the patient received a total of six blood transfusions and seven erythropoietin injections (4000–8000 IE). Laboratory tests for heparin-induced thrombocytopenia and thrombotic thrombocytopenic purpura were negative. Liver enzymes were elevated (Figure 1(c)), and we detected anti-mitochondrial M2 antibodies at a low titer with 22 IU/mL being compatible with autoimmune hepatitis. In January 2020, filgrastim was injected since neutrophil count declined to 240 cells per µL. Complement factor I was profoundly decreased with 1 µg/mL (normal range, 18–48 µg/mL) indicating increased complement activation. Genetic analyses did not unveil any mutation within the complement system. In order to reduce complement activation, treatment with eculizumab was commenced in December 2019. Hemolysis and thrombocytopenia resolved, but the patient remained dialysis dependent (Figure 1(a)).

## Discussion

In anti-GBM glomerulonephritis, circulating autoantibodies raised toward the noncollagenous domain of the α3 chain of type IV collagen (α3IVnc) cause rapidly progressive glomerulonephritis and often result in end-stage renal failure, as it was the case in our patient. The α3IVnc is sequestrated within hexamers formed from cross-linked α3/α5 and α4/α5 heterodimers. It is suggested that disruption of the GBM exposes epitopes of the α3 chain eliciting the induction of directly pathogenic autoantibodies.^10^ Anti-GBM disease is a rare autoimmune disorder with an incidence estimated at one patient per million population.^10^ In our patient, we did not identify any preceding infection of the urinary tract or the kidney that might have represented the above-mentioned trigger. Only the fact that cladribine is eliminated by the kidneys might suggest nephrotoxicity. Ultrastructural changes in the glomeruli of the kidney of white Wistar rats following cladribine treatment have been reported. However, this study did not find any disruption of the anti-glomerular basement membrane.^11^ Since no case of nephrotoxicity in humans has been reported to date at dosages used for MS, this etiology seems unlikely. There are several lines of evidence to suggest that T cells may have a role in anti-GBM disease. For example, in CD4^+^ and CD8^+^ knockout animals, the disease cannot be actuated in an experimental setting.^12^ There is a strong association with polymorphisms of HLA class II genes, particularly with HLA-DRB1*1501.^10^ Using transgenic mice, it has been shown that disease susceptibility is conferred by HLA-DRB1*1501 and, thus, by CD4+ cells.^13^ However, our patient was negative for HLA-DRB1*1501 excluding at least any implication of this haplotype in the current context. We suppose that the factor that conferred susceptibility was lymphopenia which is strongly associated with autoimmunity; however, it is not the only condition linked to the development of autoimmune diseases.^14^ It remains speculative whether cladribine treatment in the first year led to the development of autoreactive cells by slow lymphopenia-induced proliferation that is preferentially driven by self-antigens or whether there was a rapid spontaneous proliferation of autoreactive cells following treatment in year 2. The latter mechanism seems to require stimulation by endogenous peptide/MHC, but under conditions of severe lymphopenia it typically involves accessory signals triggered by microbes.^11^ It remains also highly speculative whether the dental infection played a role in this context. Moreover, during lymphocyte depletion, we observed a distinct increase in the relative proportion of activated T cells (Figure 1(d) and (f)). The last cladribine treatment course particularly narrowed the difference between activated (CD3^+^ HLA-DR^+^) and non-activated (CD3^+^ HLA-DR^−^) T cells suggesting that this might have caused an imbalance in disfavor of regulatory T cells resulting in decreased immune tolerance. Of note, besides the anti-GBM glomerulonephritis, our patient developed further autoimmune diseases (Coombs negative hemolysis, thrombocytopenia, and later on autoimmune hepatitis and neutropenia; Figure 1(a) and (c)) and this fact not only dampens the idea of a random coincidence, but also points toward the generation of autoreactive B cells. One can speculate that the rapid generation of autoimmunity may depend on an existing pool of autoreactive B cells that were under control prior to cladribine treatment. Indeed, several autoreactive B cells can escape from the “central tolerance” selection in the bone marrow, but after that, they acquire tolerance in the periphery by T regulatory cells or are eliminated through apoptosis.^15^ Whether the distinct lymphopenia in our patient is tantamount to a breakdown of this “peripheral tolerance” and, if at all, sufficient enough to induce autoimmunity remains open. Recent studies stress the importance of additional factors like interleukin-7 or transforming growth factor β as a sort of “second hit” for the induction of autoimmunity.^11^ We here report a case about developed autoimmunity associated with cladribine treatment. Further cases will shed light on its true incidence. Early detection is critical to prevent serious consequences. Thus, neurologists should be attentive to the development of secondary autoimmunity in cladribine-treated patients.
